# COVID- 19 in patients affected by red blood cell disorders, results from the European registry ERN-EuroBloodNet

**DOI:** 10.1186/s13023-025-03683-7

**Published:** 2025-04-16

**Authors:** Pablo Velasco Puyo, Soteroula Christou, Saveria Campisi, Maria A. Rodríguez-Sánchez, Sara Reidel, Santiago Perez-Hoyo, Miriam Mota, Irene Savvidou, Anna Rekleiti, Alessandra Salvo, Vincenzo Voi, Giovanni Battista Ferrero, Giorgia Mandrile, Carmen Maria Gaglioti, Elena Cela, Beatriz Ponce-Salas, Eduardo J. Bardón-Cancho, Pagona Flevari, Ersi Voskaridou-Dimoula, Erfan Nur, Bart J. Biemond, Polynexi Delaporta, David Beneitez-Pastor, Anna Collado Gimbert, Anna Spasiano, Tatiana Besse-Hammer, Ioannis G. Lafiatis, Laurence Dedeken, Simona Raso, Anna Ruiz-Llobet, Sabrina Bagnato, Veerle Labarque, Andreas Glenthøj, Giovan Battista Ruffo, Maria Elena Guerzoni, Kaoutar Hafraoui, Laura Pistoia, Rosamaria Rosso, Laura Tagliaferri, Paula Gonzalez-Urdiales, Fleur Samantha Benghiat, Mariane de Montalembert, Maria Jose Teles, Anna Vanderfaeillie, Elisa Bertoni, Daniela Cuzzubbo, Teresa Ferreira, Christopher J. Saunders, Eftichia Stiakaki, Ann L. Van de Velde, Michael D. Diamantidis, Jean-Louis H. Kerkhoffs, Marisa I. Oliveira, Alessandra Quota, Roberta Russo, An Van Damme, María Argüello Marina, Mikael Lorite Reggiori, Anita W. Rijneveld, Alexis Rodríguez Gallego, Raffaella Colombatti, Achille Iolascon, Ali Taher, Béatrice Gulbis, Noémi B. A. Roy, María del Mar Mañú-Pereira

**Affiliations:** 1 Pediatric Oncology and Hematology, Vall d’Hebron Barcelona Hospital, Passeig Vall Hebron 129, 08035 Barcelona, Spain; 2 Thalassaemia Clinic, Archbishop Makarios III Hospital (NAMIII), Nicosia, Cyprus; 3 Medical Area Department, ASP 8 – P.O. Umberto I, Siracusa, Italy; 4 Rare Anemia Disorders Research Laboratory, Cancer and Blood Disorders in Children, Vall d’Hebron Research Institute, Barcelona, Spain; 5 Biostatistics and Bioinformatics Unit, Vall d’Hebron Research Unit (VHIR), Barcelona, Spain; 6 Department of Clinical and Biological Science, AOU San Luigi Gonzaga, University of Turin, Turin, Italy; 7 Pediatric Hematology and Oncology Unit, Hospital General Universitario Gregorio Marañón, Universidad Complutense de Madrid, Madrid, Spain; 8 Centre of Excellence in Rare Hematological Diseases-Hemoglobinopathies, “Laiko” General Hospital of Athens, Athens, Greece; 9 Clinical Hematology, Amsterdam UMC, University of Amsterdam, Amsterdam, The Netherlands; 10 First Department of Pediatrics, Thalassemia Unit, National and Kapodistrian University of Athens, Athens, Greece; 11 Aghia Sophia” Children’s Hospital, ERN-EuroBloodNet Center, Athens, Greece; 12 Hematology Department, Hospital Universitari Vall d’Hebron, Barcelona, Spain; 13 Malalttie Rare del Globulo Rosso, AORN A. Cardarelli, Naples, Italy; 14 Clinical Research, CHU Brugmann, Brussels, Belgium; 15 Thalassemia and Sickle Cell Disease Unit, “Vostanio” General Hospital of Mytilene, Mytilene, Greece; 16 Pediatric Hemato-Oncology, Hôpital Universitaire Des Enfants Reine Fabiola, H.U.B., Brussels, Belgium; 17 Department of Hematology and Rare Diseases, V Cervello Hospital, Azienda Ospedaliera Ospedali Riuniti Villa Sofia-Cervello, Palermo, Italy; 18 Service of Pediatric Hematology, Hospital Sant Joan de Déu, Universitat de Barcelona, Institut de Recerca Hospital Sant Joan de Déu, ERN-EuroBloodNet Member, Barcelona, Spain; 19 Talasemia, Ospedale di Lentini, Lentini, Italy; 20 Department of Pediatric Hemato-Oncology, University Hospitals Leuven, Louvain, Belgium; 21 Department of Cardiovascular Sciences, Centre for Molecular and Vascular Biology, KU Leuven, Louvain, Belgium; 22 Department of Hematology, Copenhagen University Hospital – Rigshospitalet, Copenhagen, Denmark; 23 U.O. Ematologia Con Talassemia, ARNAS Civico di Cristina Benfratelli, Palermo, Italy; 24 Pediatrics Unit, Department of Medical and Surgical Sciences for Mother Children and Adults, University of Modena and Reggio Emilia, Modena, Italy; 25 Hematology, CHU Liège, Liège, Belgium; 26 Department of Radiology, Fondazione G. Monasterio CNR - Regione Toscana, Pisa, Italy; 27 UOSD Talassemia, AOU Policlinico “G. Rodolico - San Marco”, Catania, Italy; 28 Department of Pediatric Oncology, Hematology and Immunology, Hopp-Children’s Cancer Center (KiTZ) Heidelberg, University of Heidelberg, Heidelberg, Germany; 29 Pediatric Oncology and Hematology Department, Hospital Universitario Cruces, Barakaldo, Spain; 30 Clinic of Red Blood Cell Pathologies, Hematology Unit, Hôpital Erasme, Brussels, Belgium; 31 Reference Centre for Sickle Cell Disease, Department of General Pediatrics and Pediatric Infectious Diseases, Necker-Enfants Malades Hospital, Assistance Publique-Hôpitaux de Paris (AP-HP), Université Paris Cité, Paris, France; 32 Laboratory Hematology, Centro Hospitalar Universitario de Santo Antonio, Porto, Portugal; 33 Pediatrics, UMC St Pierre, Brussels, Belgium; 34 Pediatric Onco-Hematology and Bone Marrow Transplant Unit, Children’s Hospital, ASST Spedali Civili, Brescia, Italy; 35 HSCT and Cellular Therapy Unit, Department of Paediatric Haematology-Oncology, “Anna Meyer” Children’s Hospital, Florence, Italy; 36 Pediatric Department, Hospital Fernando Fonseca, Amadora, Portugal; 37 Haematology Department, Hospital Santo António Dos Capuchos - Centro Hospitalar Universitário Lisboa Central, Lisbon, Portugal; 38 Department of Pediatric Hematology-Oncology and Autologous Hematopoietic Stem Cell Transplantation Unit, University Hospital of Heraklion, University of Crete, Heraklion, Greece; 39 HematologyAntwerp University Hospital, University of Antwerp, Edegem, Belgium; 40 Department of Haematology, Thalassemia and Sickle Cell Disease Unit, General Hospital of Larissa, Larissa, Greece; 41 Hematology, HagaZiekenhuis, The Hague, The Netherlands; 42 Pediatric Hematology Unit, Hospital D. Estefânia, ULS S. José, Lisbon, Portugal; 43 Medicine Department, UOSD Talasemia Vittorio Emanuele Hospital, Gela, Italy; 44 Department of Molecular Medicine and Medical Biotechnology, University of Naples Federico II, Naples, Italy; 45 CEINGE - Biotecnologie Avanzate Franco Salvatore, Naples, Italy; 46 Department of Pediatric Hematology and Oncology, Saint Luc University Hospital, Brussels, Belgium; 47 Hematology, Hospital Universitario Príncipe de Asturias, Madrid, Spain; 48 Pediatric Oncology Unit, Pediatric Service, Son Espases Hospital, Palma, Spain; 49 Hematology, Erasmus MC, Rotterdam, The Netherlands; 50 Ethics Committees Support Unit, Vall d’Hebron Research Institute, Barcelona, Spain; 51 Clinical Pharmacology Service, Hospital Universitari Vall d’Hebron, Barcelona, Spain; 52 Department of Women’s and Children’s Health, Azienda Ospedale-Università di Padova, Padua, Italy; 53 Division of Hematology and Oncology, Department of Internal Medicine, American University of Beirut Medical Center, Beirut, Lebanon; 54 Hôpital Universitaire de Bruxelles, Université Libre de Bruxelles, LHUB-ULB, Brussels, Belgium; 55 Oxford University Hospitals NHS Foundation Trust, Oxford, UK; 56 University of Oxford, Oxford, UK

**Keywords:** COVID- 19, Red blood cell disorders, Thalassemia, Sickle cell disease, Europe

## Abstract

**Background:**

Despite several publications covering patients from multiple centers, no international registry covered all patients with red blood cell diseases (RBCD) affected by COVID- 19. The ERN-EuroBloodNet's registry provided real-time registration of SARS-CoV- 2 patients with RBCD, promoting timely disease-specific knowledge sharing during the pandemic's early stages.

**Procedures:**

The study evaluated patient distribution, the infection across different RBCDs, and severity risk factors across similar healthcare systems, using data collected from the ERN-EuroBloodNet's REDCap platform.

**Results:**

From April 2020 to April 2023, 681 infections were recorded among 663 patients, of which 373 had transfusion-dependent thalassemia or non-transfusion-dependent thalassemia (TDT/NTDT), and 269 had sickle cell disease (SCD). SCD patients had a higher incidence of COVID- 19 than those with TDT/NTDT (10.5 vs. 4.8 COVID/100 patients). Notably, 92% of the cases were mild, with neither age nor the specific RBCD affecting severity. The number of comorbidities, notably obesity and hypertension, that patients had prior to infection was associated with more severe COVID- 19. During the infection, the presence of vaso-occlusive crises, acute chest syndrome, kidney failure, and ground-glass opacities on chest tomography scans were associated with a more severe clinical picture. The vaccination rate (32%) mirrored that of the general population and showed a protective effect against severe COVID- 19. The observed mortality rate was 0.7%, aligning with Europe's general population.

**Conclusion:**

SARS-CoV- 2 infection in SCD and TDT/NTDT patients is mild and without higher mortality than the general population. The ERN-Eurobloodnet’s registry collaborative structure exemplifies the power of international cooperation in tackling rare diseases, especially during health emergencies.

**Supplementary Information:**

The online version contains supplementary material available at 10.1186/s13023-025-03683-7.

## Introduction

Upon the onset of the SARS-CoV- 2 pandemic in Europe in March 2020, our lives were significantly impacted, prompting the consideration of adapting patient management strategies to this new scenario. Early data indicated that comorbidities, including diabetes, heart disease, pulmonary hypertension, and reduced kidney or liver function, exacerbate the effects of COVID- 19. Many of these complications are prevalent in patients with red blood cell disorders (RBCD), particularly sickle cell disease (SCD) and transfusion-dependent thalassemia or non-transfusion-dependent thalassemia (TDT/NTDT), which was thought to put this patient group at higher risk for severe COVID- 19-related outcomes [1, 2].

At the time, there was a lack of existing literature on this topic making it urgent to create a centralized repository for patients with both a RBCD and COVID- 19, and to facilitate informed medical decision-making across Europe. Consequently, on April 7, 2020, the European Reference Network on Rare Hematological Diseases (ERN-EuroBloodNet) (https://eurobloodnet.eu/) came up with a European collaborative platform to gather and share real-time clinician experiences. It included updated data and weekly graphs, with the goal of rapidly identifying COVID- 19's impact on patients with RBCD and guiding their management (study protocol, VHI-ERN-2020 - 00) (Supplement 1).

Since then, numerous publications, including national registries, systematic reviews, and multicentric SCD and thalassemia registries have provided data on this topic [3–7]. However, due to the non-interoperable data sources for RBCD patients, determining the incidence of COVID- 19 and conducting analyses of outcomes using systematic reviews from reported cases is challenging.

Retrospective studies indicated a variable COVID- 19 incidence in SCD and TDT/NTDT patients, ranging from 0 to 2.4%. Severity was generally mild in SCD and TDT/NTDT patients, though a minority of cases were severe [8–15].

Mortality rates also varied, falling between 0 and 10% for SCD and 0–20% for TDT/NTDT [16–18].

The objective of the study was to describe the incidence and progression of COVID- 19 in RBCD patients across various European countries and to identify risk factors for severe infection.

## Materials and methods

### Study population

The study population included pediatric and adult patients living in Europe with confirmed COVID- 19 and affected by red blood cell disorder.

Forty-three centers from 10 European countries registered patients on the platform. For the calculation of incidence, 40 centers were included from 8 countries (Belgium, Cyprus, Denmark, Greece, Italy, Portugal, Spain, and The Netherlands) for which data on the total RBCD population were available. Centers that did not systematically report all patients’ infections were excluded.

### Data collection

The RBCD—COVID- 19 European collaborative registry platform was built using REDCap [19] and pseudo anonymized data.

Data set elements included demographics, RBCD-related data and COVID- 19-related data.

For COVID- 19 disease severity classification, the initial WHO ordinal scale and SECURE-SCD scale registry were used to summarize symptoms and supportive care in order to standardize variables from international registries for further analysis [4, 20]:

- Grade 1: Asymptomatic
- Grade 2: Mild—symptoms of acute upper respiratory tract infection, fever, fatigue, myalgia, gastrointestinal symptoms
- Grade 3: Moderate—pneumonia, with or without clinical symptoms, no hypoxia.
- Grade 4: Severe—hypoxia (O_2_ saturations less than 92%).
- Grade 5: Critical—Acute respiratory distress syndrome (ARDS), respiratory failure, encephalopathy, shock, coagulopathy, multi-organ impairment (lung, heart, kidney and/or brain) that may be life-threatening.

To analyze risk factors for severe COVID- 19, grades 1, 2, and 3 were grouped as mild, while grades 4 and 5 were classified as severe. This distinction was made based on oxygen treatment, a variable that reflects severity more specifically and homogeneously than others, such as hospitalization (initially indicated for preventive monitoring in some patients) or clinical diagnosis of pneumonia (not always confirmed by radiological images).

Vaccinations data were added in 2021 when implemented. Physicians were requested to retrospectively review their patients'vaccination status at the time of the COVID- 19 event.

The processing of personal data was conducted in full compliance with Regulation (EU) 2016/679.The Research Ethics Committee of Vall d'Hebron Hospital has confirmed that this exceptional case justifies the waiver of informed consent (Ethics Committee approval by University Hospital Vall d'Hebron dated 7 th April 2020 (PR(AG)215/2020/VHI-ERN-2020 - 00)), and it has subsequently been approved in the various countries and centers involved in the registry.

### Statistical analysis

We analyzed the descriptions of reinfections and new infections together, treating both as equivalent independent events.

Categorical variables were described using frequency and percentages, accompanied by the IC95.

Quantitative variables were described as median and interquartile range (IQR) or mean and standard deviation (SD).

Incidence was calculated as the number of COVID- 19 cases registered on REDCap platform during April 2020 and December 2022 over the population with RBCD registered in the Rare Anemias Disease Platform (RADeep) registry [21] (https://www.radeepnetwork.eu) by the centers participating in this study at the same date. Results are presented as the mean number of COVID- 19 cases/100 RBCD patients.

To compare results between RBC disorders groups (TDT/NTDT vs SCD) Mann–Whitney U test was used for quantitative variables and Chi-Square test (or Fisher’s exact test in case of frequencies below five) was used for the categorical ones.

Differences between COVID- 19 severity groups (mild vs severe COVID- 19) were also evaluated. Kruskal–Wallis test was used for quantitative variables and Chi-Square test (or Fisher’s exact test in case of frequencies below five) was used for the categorical ones.

Correlation between days at intensive care unit (ICU) and age, RBCD, presence/absence and number of comorbidities or splenectomy was tested using a Student’s t-test or Mann–Whitney U test in two categories variables and ANOVA or Kruskal–Wallis test in those variables with more than two categories.

In quantitative variables, Spearman or Pearson correlation test was used.

The distribution of COVID- 19 severity according to the patient’s age and RBCD was evaluated through descriptive analysis. The presence of differences between the ages of the patients being part of each group of RBCD was evaluated using a Kruskal–Wallis test. Differences were evaluated in all patients and separated according to COVID- 19 severity. A post-hoc Dunn’ test was performed to assess the age differences of patients with specific disorders.

Missing data has not been imputed.

## Results

### Patient distribution

Between April 2020 and April 2023, 681 infections were registered in 663 patients. Distribution of patients by RBCD are reported in Table 1.

**Table 1:** Disease group distribution of patients with SARS-COV- 2

Disease group	Diagnosis	Total N (%)
SCD	SCD SS	189 (27.8)
	SCD SC	39 (5.7)
	SCD SB0	19 (2.8)
	SCD SB +	22 (3.2)
	SCD TOTAL	**269 (39.5)**
TDT/NTDT	TDT/NTDT Major	288 (42.3)
	TDT/NTDT Intermedia	85 (12.5)
	TDT/NTDT TOTAL	**373 (54.8)**
Other RBCD	Diamond–Blackfan anemia (DBA)	4 (0.6)
	Congenital dyserythropoietic anemia type II (CDAII)	1 (0.1)
	Elliptocytosis	2 (0.3)
	Glucose- 6-phosphate dehydrogenase deficiency (G6PD)	3 (0.4)
	High Affinity Hemoglobin	1 (0.1)
	Unstable Hemoglobin	1 (0.1)
	Pyruvate Kinase Deficiency (PKD)	3 (0.4)
	Hereditary spherocytosis	24 (3.5)
	Other RBC TOTAL	**39 (5.7)**

In bold, the total number of each subgroup is highlighted: SCD, TDT/TNDT, and other RBCD

SCD: Sickle cell disease. TDT/NTDT: transfusion-dependent thalassemia or non-transfusion-dependent thalassemia. Other RBCD: Other red blood cell disorders

The distribution according to pediatric (under 18 years) or adult age groups was almost equal among patients with SCD (50.6% pediatric versus 49.4% adults), while it was predominantly adult in TDT/NTDT (9.2% pediatric versus 90.8% adults).

There were 4 registered reinfections in patients with SCD and 14 in TDT/NTDT, all of which were mild.

**Fig. 1:**
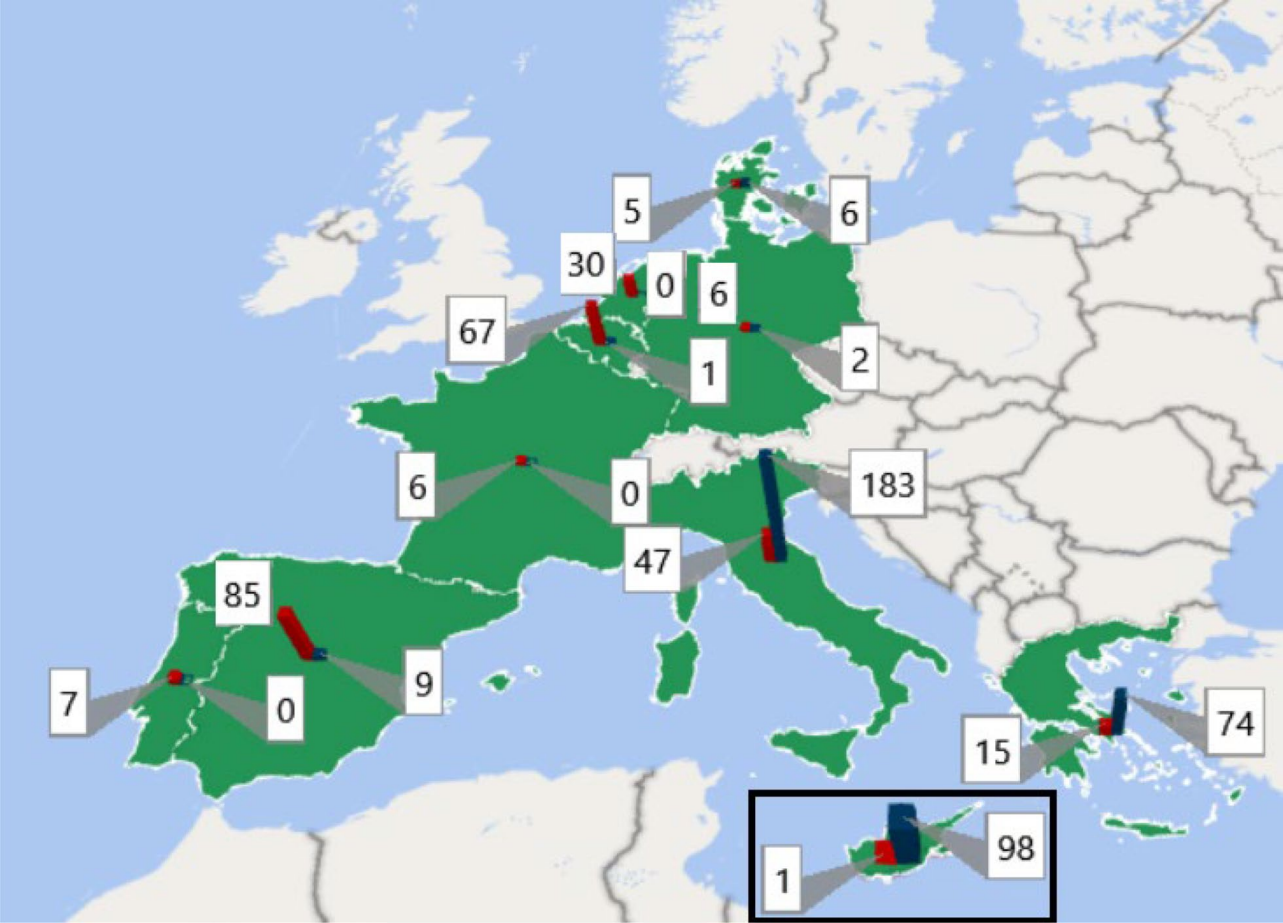
Distribution of the SARS-CoV- 2 infections by country. A total of 43 centres from 10 European countries are included in the registry (Belgium, Cyprus, Denmark, France, Germany, Greece, Italy, Portugal, Spain and The Netherlands). Red bars represent infections in SCD patients and blue TMI patients’s infections

Figure 1 depicts the distribution according to the number of TDT/NTDT and SCD patients registered.

**Fig. 2:**
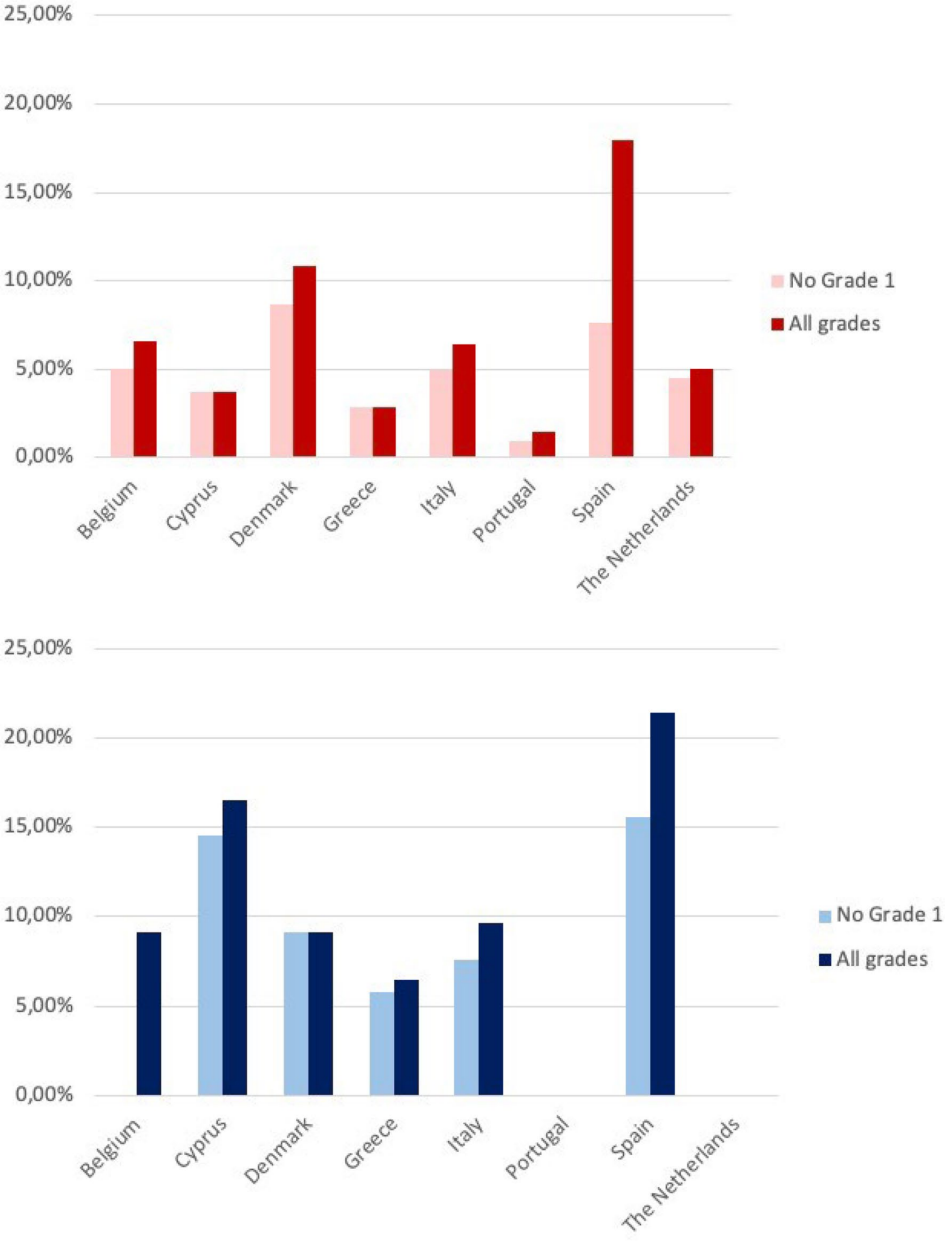
Incidence of SARS-CoV- 2 infections in SCD and TMI patients by country. Data from the RBCDs—COVID- 19 European collaborative registry on 642 patients with RBCDs and SARS-CoV- 2 infection, recorded among 7713 patients with SCD and TDT/NTDT who were monitored at centers participating in the RADeep registry during that period.The lighter bars represent symptomatic infections (not grade 1) and the darker ones represent all grades of infection, including asymptomatic ones. The color red represents infections in SCD and the color blue in TMI

The calculated incidence of symptomatic (> grade 1) COVID- 19 infection was 4.8 COVID- 19/100 SCD patients under follow-up [3.08–6.52] and 10.5 COVID- 19/100 TDT/NTDT patients under follow-up [6.69–14.31] (Fig. 2).

The incidence was lower in the pediatric population for both SCD (3.6 COVID- 19/100 SCD pediatric patients under follow-up [1.45–5.75]) versus 5.1 COVID- 19/100 SCD adult patients under follow-up [3.40–6.80]) and TDT/NTDT (7.6 COVID- 19/100 TDT/NTDT pediatric patients [0.08–15.12] versus 10.3 COVID- 19/100 TDT/NTDT adult patients [7.20–13.40]).

**Fig. 3:**
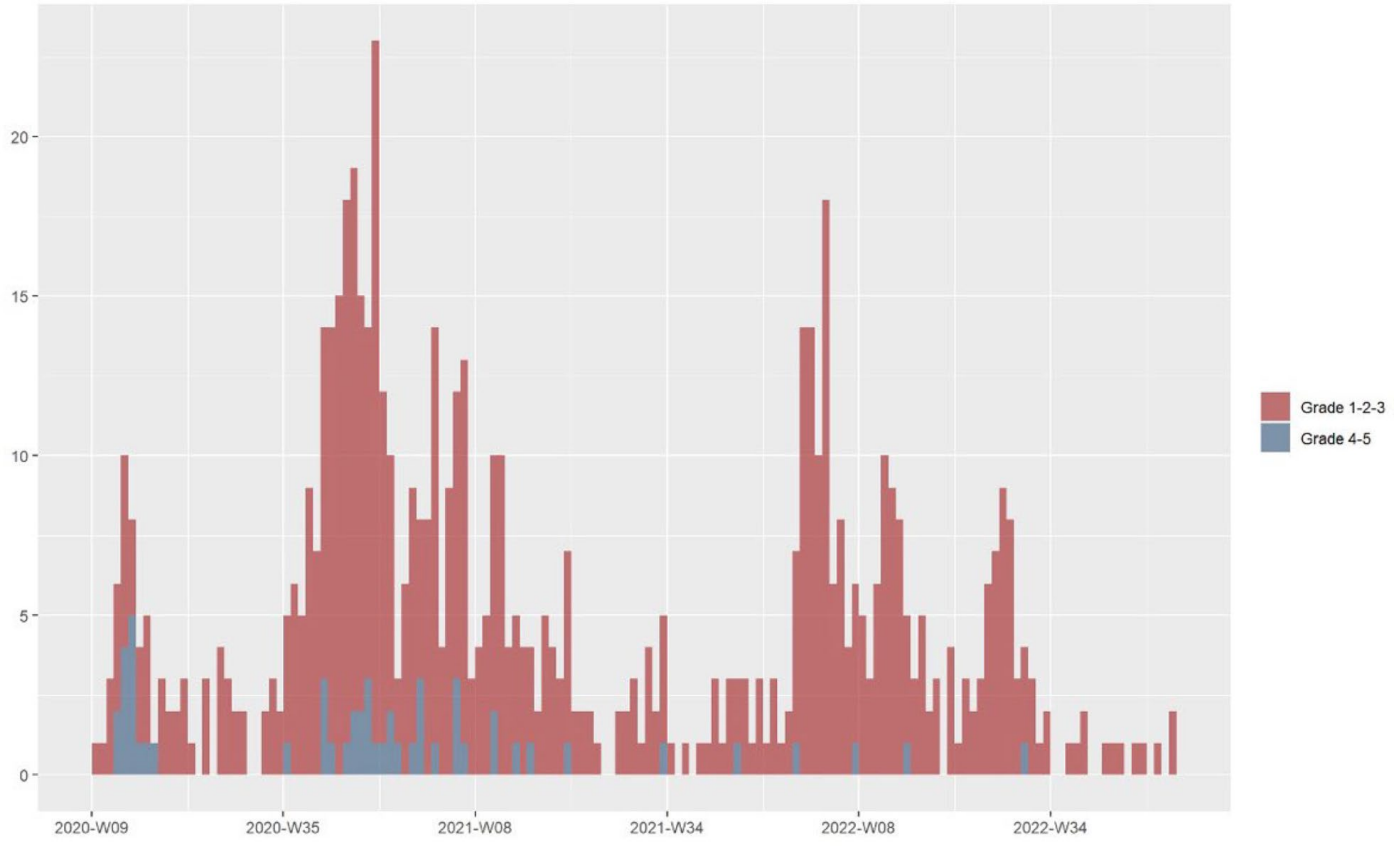
Weekly evolution of COVID- 19 Incidence and Severity in Patients with RBCDs Over Time. Weekly incidence and severity of registered patients with RBCDs over time. The blue color represents severe cases, while the red color indicates mild cases

Analyzing the evolution of the incidence and severity of registered patients, we can observe the same waves and variants as those described by the WHO [22]. The first wave had a higher proportion of severe cases (Fig. 3).

#### Description of SARS-CoV- 2 infection and comparison between patients with SCD and TDT/NTDT

Comparative analysis of RBCD baseline characteristics, COVID- 19 symptoms and outcome was performed between the two RBCD major groups, TDT/NTDT and SCD, accounting for 54.8% and 39.5% of RBCD respectively. Results are shown in Tables 2 and 3.

**Table 2:** Characteristics of SCD and TDT/NTDT patients with COVID- 19

	SCD	TDT/NTDT	All	*p*	n^α^
Age (years)	22.2 (16.7)	41.3 (14.2)	33 (18.2)	< 0.001	620
BMI	20.7 (4.4)	23.6 (4.2)	22.4 (4.6)	< 0.001	543
Ferritin level (ng/ml) *	474.5 (686.1)	1238 (1654.5)	917.1 (1422.1)	< 0.001	510
LDH (U/L) *	447.2 (204.2)	346 (200.5)	396.9 (208)	< 0.001	424
C-reactive protein (mg/dL)	3.3 (10.9)	2.3 (10.4)	3 (10.6)	0.04	210
*Treatment*					
Antibiotic Prophylaxis *	69 (38.5%)	3 (1.4%)	72 (18.1%)	< 0.001	398
Crizanlizumab	7 (3.9%)	0 (0%)	7 (1.8%)	0.003	398
Chronic transfusion (simple transfusion) *	4 (2.2%)	83 (37.9%)	87 (21.9%)	< 0.001	398
Chronic transfusion (RCE)	20 (11.2%)	2 (0.9%)	22 (5.5%)	< 0.001	398
Deferasirox *	4 (2.2%)	91 (41.6%)	95 (23.9%)	< 0.001	398
Deferiprone	2 (1.1%)	85 (38.8%)	87 (21.9%)	< 0.001	398
Deferoxamine *	1 (0.6%)	83 (37.9%)	84 (21.1%)	< 0.001	398
Hydroxyurea *	124 (69.3%)	8 (3.7%)	132 (33.2%)	< 0.001	398
Luspatercept	0 (0%)	6 (2.7%)	6 (1.5%)	0.035	398
Splenectomy *	43 (16.3%)	163 (45.5%)	206 (33.1%)	< 0.001	622
Comorbidities *	113 (43.6%)	195 (54.5%)	308 (49.9%)	0.01	617
ACS	25 (9.3%)	0 (0%)	25 (3.9%)	< 0.001	642
Arrhythmia	1 (0.4%)	32 (8.6%)	33 (5.1%)	< 0.001	642
DM *	3 (1.1%)	46 (12.3%)	49 (7.6%)	< 0.001	642
Liver cirrhosis	1 (0.4%)	16 (4.3%)	17 (2.6%)	0.005	642
ANH	8 (3%)	0 (0%)	8 (1.2%)	< 0.001	642
Smoking	1 (0.4%)	12 (3.2%)	13 (2%)	0.025	642
COVID- 19 vaccination *	29 (13.6%)	143 (44.5%)	172 (32.1%)	< 0.001	535

Descriptive analysis (mean (SD); N (%)) and p values obtained by Chi-Squared, Fisher, Mann–Whitney test or logistic regression. Only significant variables are shown

ACS: Acute chest syndrome. ANH: Avascular necrosis of the hip. BMI: Body mass index. DM: Diabetes Mellitus. RCE: Red cell exchange. SCD: Sickle cell disease. TDT/NTDT: transfusion-dependent thalassemia or non-transfusion-dependent thalassemia

^*^Significant variables once adjusted for age

^α^Available data

**Table 3:** Diagnosis, outcomes and COVID- 19 characteristics by SCD and TDT/NTDT population

	SCD	TDT/NTDT	All	*p*	n^α^
*Reason for testing*					
Screening/local protocol	56 (21.4%)	46 (12.4%)	102 (16.1%)	< 0.001	634
Clinical suspicion	126 (48.1%)	249 (66.9%)	375 (59.1%)		
Contact of known case	61 (23.3%)	75 (20.2%)	136 (21.5%)		
Travel to high-risk region	1 (0.4%)	1 (0.3%)	2 (0.3%)		
Unknown	18 (6.9%)	1 (0.3%)	19 (3%)		
*Testing method*					
Nasopharyngeal swab	183 (68%)	297 (79.6%)	480 (74.8%)	0.001	642
Oropharyngeal swab*	38 (14.1%)	14 (3.8%)	52 (8.1%)	< 0.001	642
Nasopharyngeal aspirate	6 (2.2%)	1 (0.3%)	7 (1.1%)	0.024	642
Blood (serological)*	38 (14.1%)	7 (1.9%)	45 (7%)	< 0.001	642
*Symptoms*					
Fever > 38 Cº	104(38.7%)	181 (48.5%)	285 (44.4%)	0.016	642
Cough	76 (28.3%)	149 (39.9%)	225 (35%)	0.003	642
Sore throat*	27 (10%)	93 (24.9%)	120 (18.7%)	< 0.001	642
Lethargy	11 (4.1%)	41 (11%)	52 (8.1%)	0.003	642
Rhinorrhea	26 (9.7%)	65 (17.4%)	91 (14.2%)	0.008	642
Radiology					
Chest Rx performed*	35 (21.5%)	19 (8.7%)	54 (14.2%)	< 0.001	381
Chest Rx: GGO	3 (8.6%)	8 (42.1%)	11 (20.4%)	0.01	54
Chest CT: Consolidation	9 (37.5%)	2 (8.3%)	11 (22.9%)	0.039	48
Chest CT: Pleural effusion*	8 (33.3%)	1 (4.2%)	9 (18.8%)	0.023	48
Chest CT: GGO	8 (33.3%)	20 (83.3%)	28 (58.3%)	0.001	48
Chest CT/Rx: consolidation*	15 (5.6%)	4 (1.1%)	19 (3%)	0.002	642
Chest CT/Rx: Pleural effusion*	10 (3.7%)	1 (0.3%)	11 (1.7%)	0.001	642
SOC modification due to infection	15 (6%)	48 (13.9%)	63 (10.6%)	0.003	595
Need for transfusion during COVID- 19	41 (19.1%)	144 (48.6%)	185 (36.2%)	< 0.001	511
Hospitalization *	83 (30.9%)	57 (15.3%)	140 (21.8%)	< 0.001	642
Admitted to the ICU *	12 (6%)	3 (1.3%)	15 (3.5%)	0.018	427
Severity grade					642
Grade 1	90 (33.5%)	74 (19.8%)	164 (25.5%)	< 0.001	
Grade 2	142 (52.8%)	263 (70.5%)	405 (63.1%)		
Grade 3	13 (4.8%)	11 (2.9%)	24 (3.7%)		
Grade 4	11 (4.1%)	22 (5.9%)	33 (5.1%)		
Grade 5 *	13 (4.8%)	3 (0.8%)	16 (2.5%)		

Descriptive analysis (mean (SD); N (%)) and *p* values obtained by Chi-Squared, Fisher, Mann–Whitney test or logistic regression. Only significant variables are shown

CT: Computed Tomography. GGO: Bilateral multilobar ground-glass opacification. SCD: Sickle cell disease. SOC: standard of care. TDT/NTDT: transfusion-dependent thalassemia or non-transfusion-dependent thalassemia

^*^Significant variables once adjusted for age

^α^Available data

The median age of SCD patients was lower (22.2 years) than of TDT/NTDT (41.3 years). The median age of patients with RBCD monitored in the centers participating in the RADeep registry also showed a younger median age in SCD (12.2 years) than in Thalassemia (21.1 years). To reduce the potential confounding effect of the difference in median age between SCD and Thalassemia patients, we adjusted for age when analyzing the impact of significant variables related to COVID and SCD-Thalassemia, as shown in the tables.

No differences in blood group distribution were found.

Pneumonia was diagnosed in 11% of patients, confirmed via radiology, chest X-ray, and/or computed tomography (CT) in 89% of these cases. Consolidation and pleural effusion in CT were significantly more frequently detected in SCD than in TDT/NTDT patients. In contrast, ground-glass opacification (GGO) was more commonly observed in TDT/NTDT patients via chest X-ray and CT. Considering CT as the more sensitive technique, the chest X-Ray was positive for 57% of the pleural effusions detected by CT, 46% of the consolidations, and 30% of the GGO (Table 4).

**Table 4:** Concordance between the results of chest X-ray and computed tomography imaging in patients with SCD and TDT/NTDT infected with SARS-CoV- 2, who underwent both tests

	Negative in chest X-ray	Positive in chest X-ray
*GGO*		
Negative in CT	0	7
Positive in CT	16	7
*Consolidation*		
Negative in CT	0	8
Positive in CT	5	6
*Pleural effusion*		
Negative in CT	0	1
Positive in CT	3	4

CT: computed tomography. GGO: Bilateral multilobar ground-glass opacification. TDT/NTDT: transfusion-dependent thalassemia or non-transfusion-dependent thalassemia

**Fig. 4:**
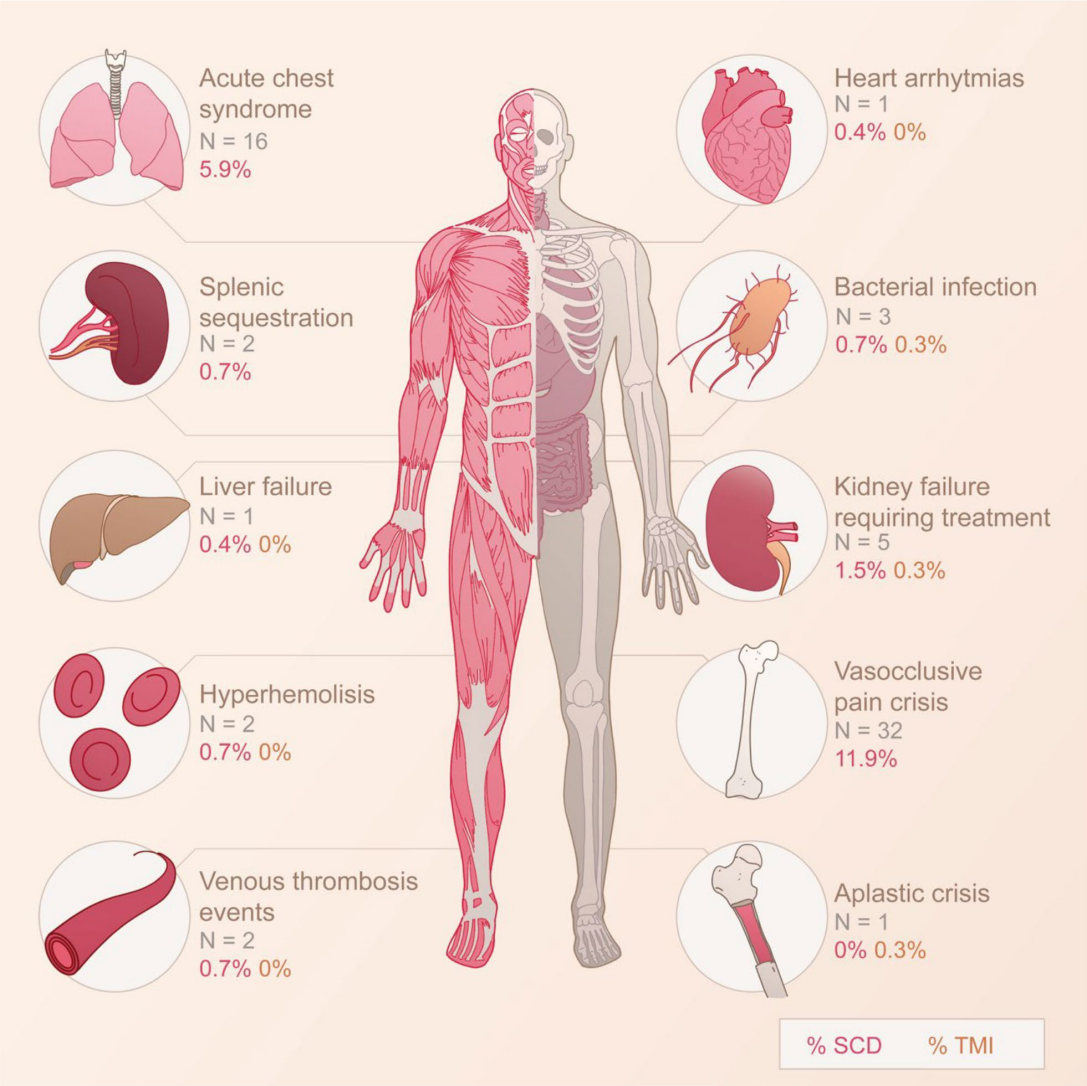
Events triggered during the SARS-CoV2 infection in SCD and TMI patients. Illustration by Jesús Sánchez.'N'represents the number of patients who experienced each event. The red percentage indicates patients with SCD who experienced the event during SARS-CoV- 2 infection, while the orange percentage indicates patients with TMI who experienced the event during SARS-CoV- 2 infection. We did not include TMI in the representation of events exclusive to SCD, such as Acute Chest Syndrome, Vasocclusive Pain Crisis, and Splenic Sequestration. SCD: Sickle cell disease. TMI: Thalasemia major o intermedia

Beyond the symptoms related to COVID- 19, we have collected data on SCD- and TDT/NTDT-related clinical events occurred during the infection (Fig. 4).

Twenty-two percent of patients were admitted to the hospital for an average stay of 8.3 days. Three-point five percent of patients were transferred to the ICU.

Three COVID- 19 related deaths were registered, all of them adults with comorbidities.

#### Severity risk factors in patients with COVID- 19 and SCD or TDT/NTDT

By consolidating the mild cases (COVID- 19 grades 1–2–3, not requiring oxygen, an indicator of severity) and the severe cases (COVID- 19 grades 4–5), we found that a significant proportion of patients with hemoglobinopathies and SARS-CoV- 2 infection experienced mild COVID- 19 (92.4%). In Table 5, we analyzed the distribution of past medical history and COVID- 19 clinical features among patients with hemoglobinopathies to identify potential risk factors for severe COVID infection.

**Table 5:** Characteristics of SCD and Thalassemia patients by COVID- 19 severity

	Grade 1–2–3 (629, 92.3)	Grade 4–5 (52, 7.6)	All	*p*	n^α^
*Baseline characteristics*					
*TDT/NTDT*					
Thalassemia major	274 (78.7%)	14 (56%)	288. (77.2%)	0.018	373
Thalassemia intermedia	74 (21.3%)	11 (44%)	85 (22.8%)		
*Age group (years)*					
0–1	3 (0.5%)	1 (1.9%)	4 (0.6%)	0.018	659
1–10	86 (14.2%)	1 (1.9%)	87 (13.2%)		
10–18	82 (13.5%)	7 (13.5%)	89 (13.5%)		
18–85	436 (71.8%)	43 (82.7%)	479 (72.7%)		
BMI*	22.2 (4.5)	25.1 (4.8)	22.4 (4.6)	< 0.001	570
Creatinine (mg/L)*	6.9 (50.1)	5.3 (12.4)	6.8 (48.4)	0.008	513
Hypertension*	18 (2.9%)	6 (11.5%)	24 (3.5%)	0.007	681
Comorbidities count per patient*	0.7 (0.9)	1 (1.1)	0.7 (1)	0.021	681
ACS in SCD *	19 (7.8%)	6 (25%)	25 (9.3%)	0.015	269
Treatment with hydroxyurea in SCD*	110 (67.1%)	14 (93.3%)	124 (69.3%)	0.04	179
Patient vaccinated prior to COVID*	171 (32.7%)	4 (11.1%)	175 (31.3%)	0.012	559
*COVID infection*					
Pneumonia	26 (4.4%)	45 (88.2%)	71 (11.2%)	< 0.001	636
Clinical	10 (1.6%)	31 (59.6%)	41 (6%)	< 0.001	681
Radiological	25 (4%)	39 (75%)	64 (9.4%)	< 0.001	681
GGO in patients with chest X-ray performed	3 (8.8%)	9 (33.3%)	12 (19.7%)	0.039	61
*Acute events during COVID*					
Co-infection*	1 (0.2%)	4 (10%)	5 (1.1%)	< 0.001	471
Bacterial co-infection *	1 (0.2%)	2 (3.8%)	3 (0.4%)	0.016	681
Kidney failure requiring treatment *	0 (0%)	6 (11.5%)	6 (0.9%)	< 0.001	681
Kidney failure requiring treatment in SCD	0 (0%)	4 (16.7%)	4 (1.5%)	< 0.001	269
ACS in SCD*	4 (1.6%)	12 (50%)	16 (5.9%)	< 0.001	269
VOC in SCD*	23 (9.4%)	9 (37.5%)	32 (11.9%)	< 0.001	269
Need for transfusion during COVID- 19	161 (32.7%)	27 (61.4%)	188 (35.1%)	< 0.001	536
Hospitalization	99 (15.7%)	50 (96.2%)	149 (21.9%)	< 0.001	681

Descriptive analysis (mean (SD); N (%)) and p values obtained by Chi-Squared, Fisher, Mann–Whitney test or logistic regression. Only significant variables are shown

ACS: Acute chest syndrome. BMI: Body mass index. GGO: bilateral multilobar ground glass opacification. SCD: Sickle cell disease. TDT/NTDT: transfusion-dependent thalassemia or non-transfusion-dependent thalassemia

^*^Significant variables once adjusted for age

^α^Available data

There were no differences in COVID- 19 severity among the overall RBCD patients or within the various genotypes of SCD, even when stratified by age. However, within the TDT/NTDT group, patients with thalassemia intermedia experienced a more severe course of COVID- 19 than patients with thalassemia major.

**Fig. 5:**
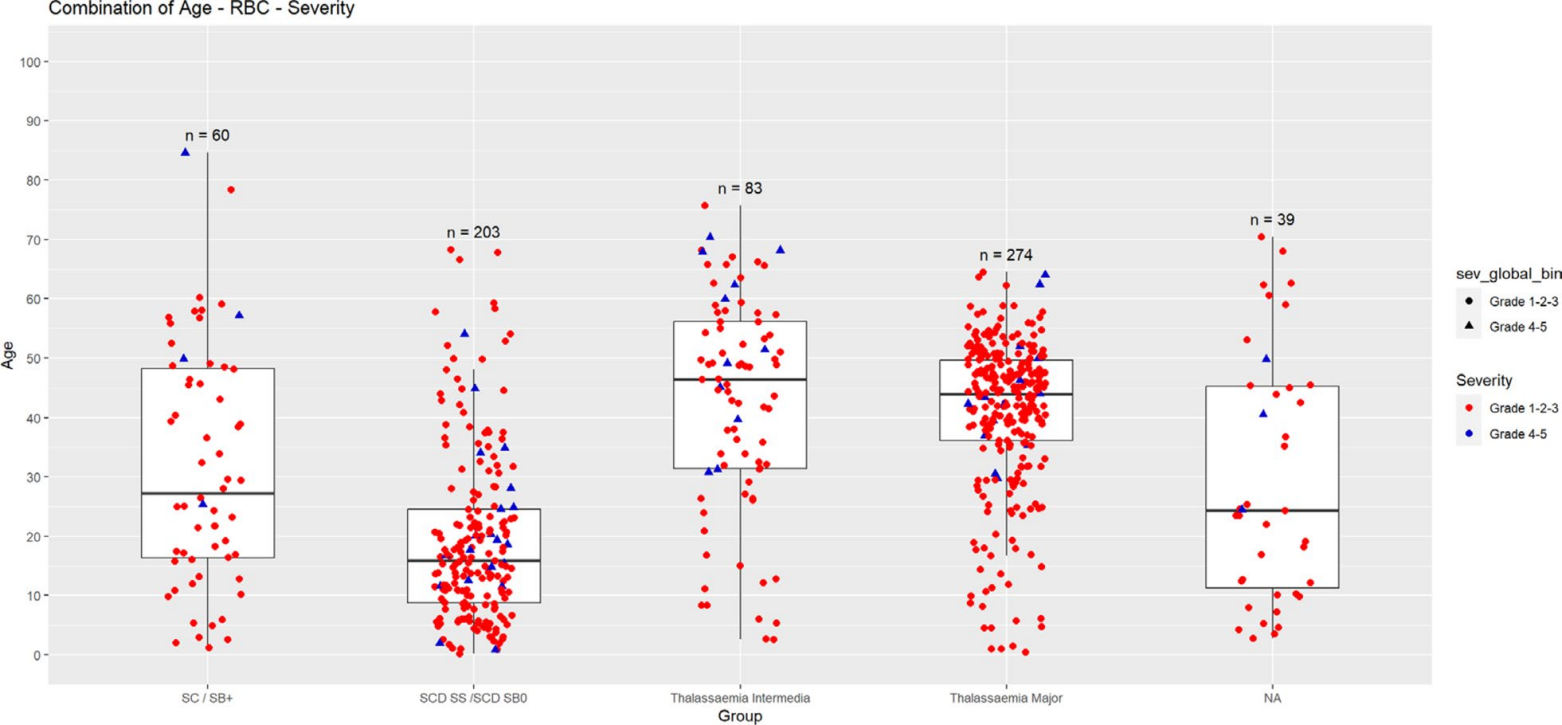
Distribution of cases according to age (years) and genotype of SCD patients (severe genotype SS/SB0 and mild genotype SC/SB +) and phenotype of TMI (Thalassemia Major and Intermedia). The blue triangles represent severe cases and the red circles represent mild cases. SCD: Sickle Cell Disease. SS: Homozygous SCD. SB0: SCD with beta-zero thalassemia. SB + : SCD double heterozygote with beta-plus thalassemia. SC: SCD double heterozygote with hemoglobin S and C. TMI: Thalassemia Major and Intermediate

Although we found no difference in severity by age, neither between children (< 18 years) nor adults, there was a difference between age groups, with milder symptoms in patients aged between 1 and 10 years (Table 5). Nevertheless, when comparing the distribution of mild or severe COVID- 19 according to the genotype of SCD and phenotype of TDT/NTDT, we noticed that those infected were significantly younger in the SS/SB0 group, for both mild and severe cases. The average age in severe cases was 21.3 (13.0) years for SS/SB0, 54.2 (24.4) years for SC/SB +, 52.4 (14.5) years for thalassemia intermedia, and 44.2 (10.3) years for thalassemia major (Fig. 5).

No differences were found according to gender, baseline ferritin level, LDH, bilirubin, C-reactive protein. There were no differences in the distribution of patients’ blood groups between the mild and severe COVID- 19 groups (p = 0.64).Although the creatinine levels are significantly higher in the mild COVID- 19 group, this difference is not clinically relevant (a median of 0.7 mg/L [0.5–1.6] in mild cases and 0.9 mg/L [0.6–5.1] in severe COVID- 19). Furthermore, the protein/creatinine ratio showed no significant differences between the two groups.

Previous treatments with angiotensin-converting enzyme inhibitors (n = 16), antibiotic prophylaxis (n = 72), Crizanlizumab (n = 7), chronic top-up transfusion program (neither with simple transfusion (n = 90) nor with red cell exchange (n = 22)), iron chelators (n = 272), L-glutamine (n = 1), Luspatercept (n = 6), Mitapivat (n = 1), steroids (n = 5) or other immunosuppressive drugs (n = 5), did not show an effect on COVID- 19 severity. There were more patients treated with hydroxyurea in the SCD group with more severe COVID- 19.

In all patients with RBCD, those with arterial hypertension experienced more severe COVID- 19, although this effect was not observed specifically in SCD patients.

In SCD patients, a history of previous ACS was associated with severity during COVID- 19, although this history was not linked with an increased occurrence of ACS during the infection.

Prior to SARS-CoV2 infection, a history of splenectomy, liver cirrhosis, leg ulcers, eye pathology (requiring intervention), avascular necrosis of the hip, hyperhemolysis, smoking, or other comorbidities such as respiratory (chronic obstructive pulmonary disease, pulmonary hypertension, asthma), cardiac (heart failure, ischemic heart disease), renal (microalbuminuria, renal dysfunction CKD 4 and 5) or neurological (haemorrhagic and ischemic stroke, silent infarct, abnormal TCD) did not prove to be predictive of a more severe COVID- 19.

There was no significant difference when comparing the percentage of patients with comorbidities in the mild group versus severe cases (47.4% vs 61.2%). However, the number of comorbidities per patient was significantly higher in severe cases (1[0.6;1.3]) than in mild ones (0.7[0.6;0.7]).

In our study, 31.3% of patients with RBCD were vaccinated prior to their COVID- 19 infection; the analysis of this group demonstrated that the vaccinated patients had a milder course of COVID- 19.

Patients with clinical or radiological pneumonia experienced a more severe infection, with the classic COVID- 19 pattern of GGO on chest X-ray being associated with the worst outcomes (Table 5).

A total of 21.9% of patients were hospitalized. In 10% of all patients with RBCD, the standard of care was modified due to COVID- 19, which was not related to the severity of the infection.

Out of all the events indicated in Fig. 4, four were associated with an increased severity of COVID- 19, including co-infections (particularly bacterial ones) and kidney failure requiring treatment in the overall population and among SCD patients (though not in TDT/NTDT patients). Additionally, in SCD patients, ACS and VOC episodes were more common in those with severe COVID- 19. Episodes of VOC during COVID- 19 occurred more frequently in younger individuals (average age of 23 years in patients with VOC vs 33 years in patients without VOC; p 0.003), with higher baseline CRP, (C-reactive protein) (10.8 mg/dl vs 8.2 mg/dl, *p* < 0.0001) and higher steady state bilirubin levels (3 mg/dl vs 2 mg/dl, p 0.045). Transfusion during COVID- 19 was more frequent in severe than in mild cases (61.4% vs 32.7%).

Two patients met the criteria for Cytokine Release Syndrome (CRS), and none met the criteria for multisystemic inflammatory syndrome-children(MISC).

A total of 3.7% of patients required ICU admission, with a significantly different distribution depending on the RBCD group (*p* = 0.013), primarily composed of patients with SCD (70.6%), then TDT/NTDT (17.6%), and finally other RBCD (11.8%). No differences were found in the length of hospital stay based on age, sex, RBCD, comorbidities, or a history of splenectomy.

## Discussion

The platform for registering patients with SARS-CoV- 2 infection and RBCD within ERN-EuroBloodNet has been an innovative resource at the beginning of the pandemia designed to provide a rapid response to the need to share knowledge about the disease in real time.

To disseminate these data at the European and international level, internal data analyses were presented at the American Society of Hematology (ASH) 2021 and European Hematology Association (EHA) 2022 conferences [23, 24].

After the pandemic ended and detection policies relaxed, patient detection and registration decreased, signaling the end of its usefulness following the initial three years of SARS-CoV- 2. We now analyze these data. The compilation of these patients into a single harmonized registry reduces the divergences or the literature reviews [25].

This registry includes 681 infections in 663 patients with RBCD, this represents the largest European registry encompassing all RBCD groups with SARS-CoV- 2 infection, published to date.

The incidence of COVID- 19 was 4.8 COVID- 19/100 SCD patients and 10.5 COVID- 19/100 TDT/NTDT patients (Fig. 2). Similar prevalence figures were recorded in Italy, with a prevalence of 5% in SCD patients, 6.8% in TDT, and 3.3% in NTDT [3]. This is lower than the incidence in the general population in Europe, which accounts for 23–33% of cumulative incidence up until April 2023, even including the asymptomatic patients in our registry. This discrepancy may be attributed to stricter adherence to prevention strategies among individuals with RBCD, given the initially presumed higher risk of severe COVID- 19 in this population. The majority of patients had mild COVID- 19 (92%) as other registries have described [4, 6, 26]**.** This may be due to being a more studied population upon presenting symptoms compatible with the infection, especially in the early stages of the pandemic.

Comparing the evolution of the incidence and severity of registered patients with the distribution of variants described during the pandemic, we can infer a similar trend with respect to the variants in patients with RBCD [22]. The first wave was more aggressive (March 2020 to June 2020), the second wave (September 2020 to February 2021) had a higher incidence coinciding with improved diagnostic capacity, and the wave during the period of the Omicron variant (December 2021 to March 2022) was the mildest as shown in other studies [26–28]. Furthermore, the pronounced severity of the initial wave can likely be attributed to reduced population immunity. This is supported by the observation of milder cases in our cohort during subsequent reinfections or post-vaccination, which further supports the recommendation of vaccination for patients with RBCD [6]. Indeed, the vaccination rate was 32%, similar to the 35% rate in the general population in Europe [22].

COVID- 19 was more frequent in adults with TDT/NTDT, but equally distributed in children and adults with SCD, as other registries have shown [4]. While age has not been proven to be a risk factor for severe COVID- 19 in RBCD, patients aged between 1 and 10 years had milder COVID- 19. On the other hand, severe cases in more aggressive SCD genotypes (SS/SB0) occurred at a younger average age (21.3 years) as has been observed in other registries [5, 29]. This highlights the importance of implementing the same vaccination and prevention strategies in the pediatric population as in adults, particularly within the SCD patient group.

Some registries have highlighted that age, BMI and comorbidities in SCD, such as heart conditions (including arterial hypertension), lung and renal diseases, prior VOC or ACS, or not been treated with hydroxyurea, were related to severe COVID- 19 [3–5, 30]. Although an association between COVID- 19 severity and blood group A was initially suspected early in the pandemic, we did not find a relationship between blood group and COVID- 19 severity, as recent publications have confirmed [31].

Among the risk factors for more severe infection, we found that the number of comorbidities per patient influenced the severity of COVID- 19, as shown in other studies [3]. Among them, the comorbidities that proved to act as independent factors in the outcome of the infection were BMI and arterial hypertension in RBCD patients, and hydroxyurea treatment in SCD patients. Indeed, these factors remained related to severity after adjusting for age. The role of hydroxyurea treatment during COVID- 19 has been controversial in various studies and inconclusive in systematic reviews [25]. The high percentage of patients treated with hydroxyurea in severe cases might suggest that this specific population had a more active SCD.

In general, no RBCD group presented a higher risk of severe COVID- 19, not even within the different SCD genotypes. While there have been studies suggesting that the SC genotype might experience more severe COVID- 19, other studies have not found a relationship between genotype and the severity of COVID- 19 [18, 30, 32, 33]. This is supported by an analysis of the National Inpatient Sample records of 102,975 COVID- 19 hospitalizations with SCD [29]. In previous publications, more severe COVID- 19 cases have been reported in NTDT than in TDT [7, 34]. Although we did not observe this association, we did note a trend toward more severe infections in thalassemia intermedia genotypes compared to those with thalassemia major, which may be related to more comprehensive monitoring and earlier detection in the latter group.

The presenting symptoms were similar to those of the general population. Eleven percent presented with pneumonia, consistent with other registries reporting incidences between 16 and 27% [1, 2], often showing the classic COVID- 19 ground-glass opacities (GGO) on CT, which were associated with more severe COVID- 19, in contrast to consolidation and pleural effusion.

Middle age, renal and liver failure, vasopressor use, and shock have been described as predictors of in-hospital mortality in patients with SCD [29]. In our study, VOC and ACS events during infection were associated with more severe COVID- 19. VOC was related to younger age and a more active steady state of SCD, with higher levels of CRP and bilirubin, but ACS was not related to a history of ACS, as described in other studies [33]. The onset of kidney failure requiring treatment, and the presence of another infection, particularly bacterial ones, also indicated greater severity. Transfusion during COVID- 19 was associated with more severe cases, being more frequent in TDT/NTDT than in SCD patients.

Twenty-two percent of patients were hospitalized, lower than described in other registries [6] but similar to Italian data [3], with an average stay of 8.3 days. Three-point seven percent were admitted to the ICU. There were three deaths, all with comorbidities, accounting for a 0.7% mortality rate, which is lower than that described in initial registries, when there was less vaccination rate [4, 30, 35], and similar to the 0.8% rate in the general population in Europe [22]. The mortality rate has been described higher in several publications with a broad heterogeneity of data, ranging from 3.2 to 8.4% [25]. However, long-term follow-up studies have shown that SARS-CoV- 2 did not result in higher mortality in SCD or TDT/NTDT patients, and that the mortality rate of these patients with COVID- 19 is similar to that of non-SCD and non-TDT/NTDT patients [7, 36–38]. Nevertheless, this low impact on mortality in SCD and TDT/NTDT patients could be a result of the close and exhaustive care given to these patients. This should be specifically analyzed in countries with fewer resources, as a high-income status has been associated with favorable outcomes [29, 39, 40].

One of the limitations of this study is that the data were sourced from a voluntary registry, which could introduce a potential bias in patient inclusion. To mitigate this bias, we directly contacted the healthcare professionals responsible for the management of patients to ensure that all RBCD patients in their centers were accurately recorded during the study period.

With this broad and comprehensive analysis, we hope to have provided sufficient evidence to contribute, along with other publications and registries, to the knowledge on SARS-CoV- 2 infections in RBCD. This ERN-EuroBloodNet Platform quickly and efficiently responded to a European health emergency. Indeed, the structure of this registry, together with RADeep, has overcome all the logistical, ethical, and legal challenges at the European level, and can serve as a platform for a faster response to future health emergencies in a globalized world sensitive to pandemics.

## Supplementary Information

Additional file 1.

## Data Availability

The datasets during and/or analysed during the current study available from the corresponding author on reasonable request.

## Abbreviations

ACS: Acute chest syndrome
ARDS: Acute respiratory distress syndrome
GGO: Ground-glass opacification
RBCD: Red blood cell diseases
SCD: SCD
TCD: Transcranial doppler
TDT: Transfusion-dependent thalassemia
NTDT: Non-transfusion-dependent thalassemia

## References

[1] Farmakis D, Porter J, Taher A, Domenica Cappellini M, Angastiniotis M, Eleftheriou A. 2021 Thalassaemia international federation guidelines for the management of transfusion-dependent Thalassemia. Hemasphere. 2022;6(8): e732. 10.1097/HS9.0000000000000732. 35928543 10.1097/HS9.0000000000000732 PMC9345633

[2] Piel FB, Rees DC, DeBaun MR, et al. Defining global strategies to improve outcomes in sickle cell disease: a Lancet Haematology Commission. Lancet Haematol. 2023;10(8):e633–86. 10.1016/S2352-3026(23)00096-0. 37451304 10.1016/S2352-3026(23)00096-0 PMC11459696

[3] Longo F, Gianesin B, Voi V, et al. Italian patients with hemoglobinopathies exhibit a 5-fold increase in age-standardized lethality due to SARS-CoV-2 infection. Am J Hematol. 2022;97(2):E75–8. 10.1002/ajh.26429. 34861054 10.1002/ajh.26429 PMC9011434

[4] Mucalo L, Brandow AM, Dasgupta M, et al. Comorbidities are risk factors for hospitalization and serious COVID-19 illness in children and adults with sickle cell disease. Blood Adv. 2021;5(13):2717–24. 10.1182/bloodadvances.2021004288. 34196678 10.1182/bloodadvances.2021004288 PMC8248962

[5] Castonguay M, Dakhallah N, Desroches J, et al. COVID-19 and sickle cell disease in the province of Quebec, Canada: outcomes after two years of the pandemic. J Clin Med. 2022;11(24):7361. 10.3390/jcm11247361. 36555978 10.3390/jcm11247361 PMC9781039

[6] Mucalo L, Brandow AM, Singh A. A perspective on the sickle cell disease international COVID-19 registry. Best Pract Res Clin Haematol. 2022;35(3): 101385. 10.1016/j.beha.2022.101385. 36494148 10.1016/j.beha.2022.101385 PMC9509018

[7] El-Battrawy I, Longo F, Núñez Gil IJ, et al. Thalassaemia is paradoxically associated with a reduced risk of in-hospital complications and mortality in COVID-19: data from an international registry. J Cell Mol Med. 2022;26(9):2520–8. 10.1111/jcmm.17026. 35355397 10.1111/jcmm.17026 PMC9077285

[8] Brousse V, Holvoet L, Pescarmona R, et al. Low incidence of COVID-19 severe complications in a large cohort of children with sickle cell disease: a protective role for basal interferon-1 activation? Haematologica. 2021;106(10):2746–8. 10.3324/haematol.2021.278573. 33979992 10.3324/haematol.2021.278573 PMC8485686

[9] Telfer P, De la Fuente J, Sohal M, et al. Real-time national survey of COVID-19 in hemoglobinopathy and rare inherited anemia patients. Haematologica. 2020;105(11):2651–4. 10.3324/haematol.2020.259440. 33054122 10.3324/haematol.2020.259440 PMC7604629

[10] Argüello-Marina M, López-Rubio M, Morado M. SARS-CoV-2 infection in patients with sickle cell disease. Med Clin (Barc). 2021;157(7):345–6. 10.1016/j.medcli.2021.03.033. 34074477 10.1016/j.medcli.2021.03.033 PMC8112321

[11] Munaretto V, Voi V, Palazzi G, et al. Acute events in children with sickle cell disease in Italy during the COVID-19 pandemic: useful lessons learned. Br J Haematol. 2021;194(5):851–4. 10.1111/bjh.17546. 34036565 10.1111/bjh.17546 PMC8239759

[12] Minniti CP, Zaidi AU, Nouraie M, et al. Clinical predictors of poor outcomes in patients with sickle cell disease and COVID-19 infection. Blood Adv. 2021;5(1):207–15. 10.1182/bloodadvances.2020003456. 33570644 10.1182/bloodadvances.2020003456 PMC7802524

[13] Zafari M, Rad MTS, Mohseni F, Nikbakht N. β-Thalassemia major and coronavirus-19, mortality and morbidity: a systematic review study. Hemoglobin. 2021;45(1):1–4. 10.1080/03630269.2020.1857266. 33317358 10.1080/03630269.2020.1857266

[14] Haghpanah S, Hosseini-Bensenjan M, Sayadi M, Karimi M. Incidence rate of COVID-19 infection in Hemoglobinopathies: A systematic review and meta-analysis. Hemoglobin. 2021;45(6):371–9. 10.1080/03630269.2021.1927751. 34027786 10.1080/03630269.2021.1927751

[15] de Sanctis V, Canatan D, Corrons JLV, et al. Preliminary data on COVID-19 in patients with Hemoglobinopathies: A multicentre ICET-A study. Mediterr J Hematol Infect Dis. 2020;12(1): e2020046. 10.4084/MJHID.2020.046. 32670524 10.4084/MJHID.2020.046 PMC7340245

[16] de Thiago SV, Braga JAP, Loggetto SR. Hemoglobinopathy and pediatrics in the time of COVID-19. Hematol Transfus Cell Ther. 2021;43(1):87–100. 10.1016/j.htct.2020.11.002. 33289008 10.1016/j.htct.2020.11.002 PMC7709722

[17] Lee JX, Chieng WK, Lau SCD, Tan CE. COVID-19 and Hemoglobinopathies: a systematic review of clinical presentations, investigations, and outcomes. Front Med (Lausanne). 2021;8: 757510. 10.3389/fmed.2021.757510. 34722593 10.3389/fmed.2021.757510 PMC8549676

[18] Sayad B, Karimi M, Rahimi Z. Sickle cell disease and COVID-19: Susceptibility and severity. Pediatr Blood Cancer. 2021;68(8): e29075. 10.1002/pbc.29075. 34061431 10.1002/pbc.29075 PMC8209850

[19] Harris PA, Taylor R, Thielke R, Payne J, Gonzalez N, Conde JG. Research electronic data capture (REDCap)–a metadata-driven methodology and workflow process for providing translational research informatics support. J Biomed Inform. 2009;42(2):377–81. 10.1016/j.jbi.2008.08.010. 18929686 10.1016/j.jbi.2008.08.010 PMC2700030

[20] Dong Y, Mo X, Hu Y, et al. Epidemiological characteristics of 2143 pediatric patients with 2019 coronavirus disease in China. Pediatrics. 2020. 10.1542/peds.2020-0702. 32600305 10.1186/s12887-020-02224-4 PMC7322851

[21] Mañú Pereira MDM, Colombatti R, Alvarez F, et al. Sickle cell disease landscape and challenges in the EU: the ERN-EuroBloodNet perspective. The Lancet Haematology. 2023;10(8):e687–94. 10.1016/S2352-3026(23)00182-5. 37451300 10.1016/S2352-3026(23)00182-5

[22] WHO Coronavirus (COVID-19) Dashboard. Accessed August 3, 2023. https://covid19.who.int

[23] Velasco P, Longo F, Piolatto A, et al. S283: ERN-Eurobloodnet European registry of patients affected by red blood cell disorders and COVID-19. HemaSphere. 2022;6:184. 10.1097/01.HS9.0000844024.74005.fd.

[24] Velasco P, Longo F, Piolatto A, et al. ERN-EuroBloodNet European registry of patients affected by red blood cell disorders and COVID-19. Blood. 2021;138(Supplement 1):4058–4058. 10.1182/blood-2021-147236.

[25] Pereira LRG, da Silva MVG, Germano CMR, Estevao IF, Melo DG. Impact of the SARS-CoV-2 infection in individuals with sickle cell disease: an integrative review. Front Med. 2023. 10.3389/fmed.2023.1144226. 10.3389/fmed.2023.1144226 PMC10187638 37200963

[26] Martin OY, Margulies S, Speller-Brown B, Majumdar S, Darbari DS, Campbell AD. The evolution of the COVID-19 pandemic in paediatric patients with sickle cell disease: from Alpha to Omicron. Br J Haematol. 2023;202(3):479–84. 10.1111/bjh.18867. 37217303 10.1111/bjh.18867

[27] CoVariants: Per Country. Accessed August 3, 2023. https://covariants.org/per-country

[28] Derdevet J, Ranque B, Khimoud D, et al. Efficacy of COVID-19 vaccination in adult patients with sickle cell disease during the Omicron wave in France. Eur J Haematol. 2023;111(3):509–12. 10.1111/ejh.14034. 37380177 10.1111/ejh.14034

[29] Ilerhunmwuwa NP, Inyang L, Wasifuddin M, et al. Demographics and outcomes of hemoglobin genotype in hospitalized patients with COVID-19 and sickle cell disease in the United States. Eur J Haematol. 2023;111(4):611–9. 10.1111/ejh.14054. 37477175 10.1111/ejh.14054

[30] Arlet J, Lionnet F, Khimoud D, et al. Risk factors for severe COVID-19 in hospitalized sickle cell disease patients: a study of 319 patients in France. Am J Hematol. 2022;97(3):E86–91. 10.1002/ajh.26432. 34882837 10.1002/ajh.26432 PMC9011445

[31] Golinelli D, Boetto E, Maietti E, et al. The association between ABO blood group and SARS-CoV-2 infection: a meta-analysis. PLoS ONE. 2020;15(9): e0239508. 10.1371/journal.pone.0239508. 32946531 10.1371/journal.pone.0239508 PMC7500631

[32] Cai J, Chen-Goodspeed A, Idowu M. Risk and protective factors for severe COVID-19 infection in a cohort of patients with sickle cell disease. J Investig Med. 2022;70(5):1243–6. 10.1136/jim-2021-002196. 35260481 10.1136/jim-2021-002196

[33] Christian J, Lanzkron S, Naik RP. COVID-19 outcomes in sickle cell disease and sickle cell trait. Best Pract Res Clin Haematol. 2022;35(3): 101382. 10.1016/j.beha.2022.101382. 36494153 10.1016/j.beha.2022.101382 PMC9450487

[34] Al-Kuraishy HM, MazharAshour MH, Saad HM, Batiha GES. COVID-19 and β-thalassemia: in lieu of evidence and vague nexus. Ann Hematol. 2023. 10.1007/s00277-023-05346-8. 37405444 10.1007/s00277-023-05346-8

[35] Silva Borborema T, Moreira Brito JC, Lima Batista EM, Siqueira BR. Case fatality rate and severity of COVID-19 among patients with sickle cell disease: a systematic review and meta-analysis. Hemoglobin. 2023;47(2):85–96. 10.1080/03630269.2023.2219847. 37325879 10.1080/03630269.2023.2219847

[36] de Oliveira C, M, Soares VJ, Braga JAP, et al. The impact of COVID-19 in children with sickle cell disease: results of a multicentric registry. PLOS ONE. 2023;18(4):e0282423. 10.1371/journal.pone.0282423. 37023037 10.1371/journal.pone.0282423 PMC10079108

[37] Ilerhunmwuwa NP, Inyang L, Hakobyan N, et al. Outcomes of COVID-19 hospitalizations in patients with sickle cell disease: a nationwide analysis. Eur J Haematol. 2023;111(3):432–40. 10.1111/ejh.14024. 37290934 10.1111/ejh.14024

[38] Feit A, Gordon M, Alamuri TT, et al. Long-term clinical outcomes and healthcare utilization of sickle cell disease patients with COVID-19: a 2.5-year follow-up study. Eur J Haematol. 2023;111(4):636–43. 10.1111/ejh.14058. 37492929 10.1111/ejh.14058

[39] Atmakusuma TD. COVID-19 in patients with transfusion dependent Thalassemia (TDT) in Indonesia: characteristics of the disease and patients, and comparison between epidemiological data for COVID-19 and Thalassemia in Indonesia and Southeast Asia. Hematol Rep. 2022;14(1):2–12. 10.3390/hematolrep14010002. 35323173 10.3390/hematolrep14010002 PMC8953260

[40] Nachega JB, Sam-Agudu NA, Machekano RN, et al. Assessment of clinical outcomes among children and adolescents hospitalized with COVID-19 in 6 Sub-Saharan African countries. JAMA Pediatr. 2022;176(3): e216436. 10.1001/jamapediatrics.2021.6436. 35044430 10.1001/jamapediatrics.2021.6436 PMC8771438

