# Supplementary material for: COVID- 19 in patients affected by red blood cell disorders, results from the European registry ERN-EuroBloodNet
Source: Orphanet J Rare Dis. 2025 Apr 16;20:183. doi: 10.1186/s13023-025-03683-7 (PMC12001635; doi:10.1186/s13023-025-03683-7)
Supplement: Supplementary file 1 — Additional file 1. [file 13023_2025_3683_MOESM1_ESM.pdf]

Study Protocol

Study Title:

ERN-EuroBloodNet Registry on patients with rare red blood cell defects and COVID-19

Study Code: VHI-ERN-2020-00

Principal Investigator

Maria del Mar Mañú Pereira

ERN- EuroBloodNet Scientific Director

e-mail: mar.manu@vhir.org

Phone: +34625920129

Department: Translational Research in Child and Adolescent Cancer – Rare anaemia disorders program

Institution: Vall d'Hebron Research Institute (VHIR)

Passeig Vall d'Hebron, 119-129

8035 Barcelona, Spain

Principal Investigator

Pablo Velasco Puyó

e-mail: pvelasco@vhebron.net

Phone: +34677738028

Department: Translational Research in Child and Adolescent Cancer – Rare anaemia disorders program / Pediatric Oncology and Hematology

Institution: Vall d'Hebron Research Institute (VHIR) / University Hospital Vall d'Hebrón

Passeig Vall d'Hebron, 119-129

8035 Barcelona, Spain

Principal Investigator

David Beneítez Pastor

e-mail: dbeneitez@vhebron.net

Phone: +34616301936

Department: Hematology

Institution: Vall d'Hebron Oncology Institute (VHIO) / University Hospital Vall d'Hebrón

Passeig Vall d'Hebron, 119-129

8035 Barcelona, Spain

Scientific Committee

- Eduardo Jesús Bardón-Cancho, MD, Pediatric Hematologist, Hospital General Universitario Gregorio Marañón, Spain
- David Benéitez, Hematologist, Vall d'Hebron University Hospital, Spain
- Wilfried Blot, PhD, University Medical Center Freiburg, Germany
- Elena Cela, MD, Pediatric Hematologist, Hospital General Universitario Gregorio Marañón, Spain
- Raffaella Colombatti, MD, Pediatric Hematologist, AO Padova, Italy
- Mariane de Montalembert, MD, Pediatric Hematologist, Hôpital Necker-Enfants Malades, France
- Andreas Glenthøj, MD, Hematologist, Herlev and Gentofte Hospital, Denmark
- Béatrice Gulbis, MD, Hematologist, Hôpital ERASME-CUB, LHUB-ULB, Belgium.
- Baba Inusa, MD, Pediatric Hematologist, Guy's and St Thomas' NHS Foundation Trust Healthcare, United Kingdom
- Joachim Kunz, MD, Pediatric Hematologist, Zentrum für Kinder- und Jugendmedizin Heidelberg, Germany
- Stephan Lobitz, MD, Pediatric Hematologist, GKM Koblenz, Germany
- María del Mar Mañú Pereira, PhD, Researcher, Vall d'Hebron Research Institute, Spain
- Erfan Nur, MD, Internist-Hematologist, Amsterdam UMC, Netherlands
- Alexis Rodríguez, Clinical Pharmacologist, Master in Bioethics and Law, Member of the Research Ethics Committee of the Vall d'Hebron Hospital, Spain
- Eduard van Beers, MD, Hematologist, University Medical Center Utrecht, The Netherlands
- Pablo Velasco Puyo, MD, Pediatric Hematologist, Vall d'Hebron University Hospital, Spain

Protocol Synopsis

Study Title:

ERN-EuroBloodNet Registry on patients with rare red blood cell defects and COVID-19

Study Objectives:

Primary objective: To pool evidence on the clinical management and outcomes of patients affected by red blood cell disorders and COVID-19 for supporting daily medical practice while enabling inter-professional consultation of complex cases.

Secondary objective: To perform observational studies in the different cohorts of patients, including Sickle Cell Disease, Thalassaemia, Enzymopathies and Membranopathies patients in pediatric and adult stages.

Methodology:

Multi-centric observational study with retrospective and prospective gathering of data.

Population:

Patients both pediatric and adults with confirmed COVID19 and affected by a rare anaemia disorder due to a red blood cell defect according to ORPHANET classification: Hemoglobinopathy ORPHA68364, rare constitutional hemolytic anemia due to an enzyme disorder ORPHA98369 and rare constitutional hemolytic anemia due to a red cell membrane anomaly ORPHA98364.

1. Introduction

Due to the scarcity of patients and knowledge, rare diseases, affecting less than 1 in 2000 individuals, are the area in public health in which joint efforts among European Member States is most justified and crucial. The Directive 2011/24/EU on cross border health establishes the creation of European Reference Network (ERN) based on national recognized Centres of Expertise, aiming to tackle complex or rare diseases and conditions that require highly specialised treatment and a concentration of knowledge and resources.

The first 24 ERNs covering 24 different clinical areas were officially approved by the European Commission (EC) on December 2016 and started their activity 1st March 2017, being ERN-EuroBloodNet the ERN on Rare Hematological Disorders, www.eurobloodnet.eu .

EuroBloodNet results from a joint effort of the European Hematology Association (EHA), the European Network on Rare and Congenital Anaemias (ENERCA), the European hematology patient organisations represented in both the EURORDIS European Patient Advocacy Groups (ePAGS) and the EHA Patient Organisations Workgroup.

ERN-EuroBloodNet encompasses oncological and non-oncological rare hematological diseases including rare blood cell disorders, bone marrow failures, rare coagulation disorders, polycythemia, and myeloid and lymphoid tumors. Rare hereditary hemochromatosis was also included in our network following a request from well-established patient groups and experts.

Gathering 66 highly skilled and multidisciplinary healthcare teams in 15 Member States, and advanced specialized medical equipment and infrastructures, ERN-EuroBloodNet will facilitate concentration of resources for the design, validation and implementation of high-quality and cost-effective services aimed at facing the challenges of RHD. Involvement from the outset of patient associations will contribute to patient empowerment and keeping ERN-EuroBloodNet's patient-centred approach.

EuroBloodNet's main goal is to improve the healthcare and overall quality of life of patients with a Rare Hematological Disease by:

1) Improving equal access to highly specialized healthcare delivery for RHD by facilitating the establishment of a European cross border referral system for patients and samples through a multidisciplinary patient centred approach.
2) Enhancing the best practices in prevention, diagnosis and safe clinical care across Europe based on promotion of evidence based guidelines by pooling expertise within EuroBloodNet
3) Disseminating cutting-edge knowledge and facilitate continuous medical education in the field of RHD in collaboration with the European Hematology Association and the European School of Hematology
4) Providing inter-professional consultation by sharing of expertise and safe exchange of clinical information
5) Fostering European cooperation to support epidemiological surveillance of RHD and to gather the critical mass needed for development of innovative therapies, clinical trials and cutting-edge methodologies for diagnosis.

ERN- EuroBloodNet Registry on patients with rare red blood cell defects and COVID19 is an initiative conceived in the core of the ERN aiming at supporting medical practice of COVID19 in these patients by gathering evidence on pediatric and adult COVID-19 confirmed cases in Red Blood Cell disorders across Europe.

References

1) REGULATION (EU) 2016/679 OF THE EUROPEAN PARLIAMENT AND OF THE COUNCIL of 27 April 2016 on the protection of natural persons with regard to the processing of personal data and on the free movement of such data, and repealing Directive 95/46/EC (General Data Protection Regulation). Download in August 2017 at http://eur-lex.europa.eu/eli/reg/2016/679/oj

2) Directive 95/46/EC of the European Parliament and of the Council of 24 October 1995 on the protection of individuals with regard to the processing of personal data and on the free movement of such data. Download in August 2017 at http://eur-lex.europa.eu/legal-content/en/TXT/?uri=CELEX%3A31995L0046

3) About European Reference Networks. European Commission website section on European Reference Networks. https://ec.europa.eu/health/ern_en

4) About ERN-EuroBloodNet, the European Reference Network in Rare hematological disorders. https://www.eurobloodnet.eu/

2. Rationale

As for the general population, the presence of co-morbidities such as diabetes, heart disease, pulmonary hypertension, reduced kidney and / or liver function, worsen the effects of the COVID19 infection in patients affected by red blood cell disorders (RBCDs), especially sickle cell disease (SCD) and Thalassaemia patients. However, this group of patients affected by a chronic life threating disorder that become multi-organic over the time are likely to be at increased risk of complications from COVID-19. Patients at highest risk include the elderly (>50 in our population), those with a history of respiratory or cardiac disease and those with other co-morbidities.

Most of patients affected by RBCDs undergo splenectomy as therapeutic option to improve their level of haemoglobin concentration. Splenectomized patients, or in the case of SCD with functional hyposplenism, are more vulnerable to bacterial infections / superinfections after viral infection. Acute pulmonary syndrome (ACS) is the main cause of morbidity in SCD and is often triggered by infectious events.

Currently, there is no literature on the subject. Thus, any recommendation available comes from the experience gained with previous Coronaviruses infections. Accordingly, the correct treatment and management of infection by Coronavirus SARS-COV-2 (COVID-19) in patients affected by RBCDs may be challenging given the rapid spread of the pandemia and limited literature so far, especially in some countries.

Accordingly, there is an urgent need to pool evidence in a unique repository on patients affected by RBCDs and COVID19 in order to reach critical numbers to facilitate the medical decision making process across Europe.

3. Study Objectives

3.1. Primary objective

- To pool evidence on the clinical management and outcomes of patients affected by red blood cell disorders and COVID-19 (patients' age, diagnostic, comorbidities, COVID19 severity grade, clinical manifestations, laboratory determinations, treatments administered, days of hospitalization, days at intensive cares, outcomes, sequela, death) for supporting daily medical practice while enabling inter-professional consultation of complex cases.

3.2. Secondary objective

- To perform observational studies in the different cohorts of patients, including Sickle Cell Disease, Thalassaemia, Enzymopathies and Membranopathies patients in pediatric and or adult stages.

4. Expected Outcomes

Pooling data from patients affected by a concrete disorder is needed to understand the course of the disease and investigate on new options for diagnostic procedures and treatments improving the healthcare delivered. When it comes to rare diseases, it is frequently difficult to gather data at the national level enough for validating efficiency and safety of a new healthcare service. For this reason, European collaboration is required to give robustness to provision of heathcare services to patients with a rare disease, as it is the case for rare anaemia disorders.

As of April 2, 2020, the infection caused by SARS-Cov-2 (Covid-19) has affected over 900.000 people worldwide, causing more than 45.000 deaths. Patients with rare anaemia disorders may be vulnerable to complications from Covid-19, but evidence in this population is scarce. For this reason, when a hospital receives a patient of these characteristics, has no bibliography on which to base diagnostic and therapeutic decisions. A registry that allows referral hospitals in these pathologies to enter their data at the same time that they checks the evolution of similar patients in other centers, allows showing a global picture and in real time, necessary in these dramatic moments.

The network of hospitals that will be created from this registry will hold regular meetings to analyze the data that are being introduced and to discuss possible measures against Covid-19 based on them.

The collaboration will continue with the development of observational studies that will give the necessary evidence to make recommendations for Covid management19 in hematological patients.

By pooling information in a collaborative way we aim to quickly identify the impact of COVID19 on patients with red blood cell disorders, specially sickle cell disease and thalassaemia and to understand the impact of risk factors such as prior disease complications i.e acute chest syndrome or related therapeutic interventions i.e splenectomy or blood transfusion on health outcomes.

5. Methodology

Multi-centric observational study with retrospective and prospective gathering of data.

Inclusion criteria: Patients both pediatric and adults with confirmed COVID19 and affected by a rare anaemia disorder due to a red blood cell defect according to the following ORPHA codes for rare diseases:

Orpha_Code	Disease	Orpha_Code	Disease
**68364**	**Hemoglobinopathy**		
**275752**	**Sickle cell disease and related diseases**		
		232	Sickle cell anemia
		251355	Sickle cell disease associated with an other hemoglobin anomaly
		251359	Sickle cell-beta-thalassemia disease syndrome
		251365	Sickle cell-hemoglobin C disease syndrome
		251370	Sickle cell-hemoglobin D disease syndrome
		251375	Sickle cell-hemoglobin E disease syndrome
		251380	Hereditary persistence of fetal hemoglobin-sickle cell disease syndrome
**848**	**Beta-thalassemia**		
		231214	Beta-thalassemia major
		231222	Beta-thalassemia intermedia
		231226	Dominant beta-thalassemia
		231230	Beta-thalassemia associated with another hemoglobin anomaly
		46532	Hereditary persistence of fetal hemoglobin-beta-thalassemia syndrome
		231237	Delta-beta-thalassemia
		231242	Hemoglobin C-beta-thalassemia syndrome
		231249	Hemoglobin E-beta-thalassemia syndrome
		330032	Hemoglobin Lepore-beta-thalassemia syndrome
**846**	**Alpha-thalassemia**		
		93616	Hemoglobin H disease
		163596	Hb Bart's hydrops fetalis
**98363**	**Rare hemolytic anemia**		
**98369**	**Rare constitutional hemolytic anemia due to an enzyme disorder**		
**98364**	**Rare constitutional hemolytic anemia due to a red cell membrane anomaly**		
		822	Hereditary spherocytosis
		288	Hereditary elliptocytosis
		98365	Hereditary stomatocytosis

Patients are cared for according to their treating physician’s best judgement. They are not being subjected to any experimental treatment or examination for the purposes of this study. For the avoidance of any doubt, no clinical, instrumental, laboratory assessments, or therapeutic intervention will be performed other than those required for disease management according to local best practice. Biological samples are not foreseen to be collected.

Data set elements include:

- Demographics: country of living, sex, age (only year of birth)
- Data related to Red blood cell disorder: diagnosis, co-morbidities, treatments, splenectomy, blood transfusion requirement.
- Data related to COVID19: date and method for diagnosis, severity grade, clinical manifestations i.e. pneumonia, symptoms days, acute events, treatments, days of hospitalization, days at intensive care unit, sequela, death.

The IT solution for the registry will be developed by the Statistics and Bioinformatics Unit (UEB) at VHIR. Thus, the server hosting the database and data gathered will be allocated at VHIR. The registry will be developed using Redcap, a secure web application for building and managing online databases. Individual patients’ data will be gathered in a codified way. For the avoidance of any doubt any direct identifier of the patients such as name, surname, home address or national identity card will not be gathered.

Requests to contribute to the registry will be sent by e-mail to registry promotor and once validated a user account will be generated by UEB. Thus, access to registry is fenced by login and password.

Different permission levels will be established for each user account regarding access to data and valid actions i.e. editing

By default, and aligned with primary objective of the registry which is supporting medical practice any medical centre providing data to the registry will be able to access all codified data stored in the registry and edit only data from his/her medical center. Nevertheless, limitations to access to individual data from a given medical center may be also applied in accordance to center and / or national legislation. In these cases, data gathered will be only processed for secondary objective.

For secondary objective, data gathered will be processed according to the research protocol developed by the Scientific Committee. Scientific committee shall be expanded to new members providing data to the registry based on number of patients and consensus by Scientific Committee.

Observational studies carried out within the secondary objectives will analyze if any of the rare hematological diseases are at higher risk of developing severe covid-19, if covid-19 infection triggers complications typical of these erythropathologies and if any of the current treatments are more effective in treating covid-19 infection in this population.

Descriptive analyses will be undertaken using standard statistical methods to examine the subjects' demographics, disease characteristics and management of these disorders during the COVID19 infection.

Workplan

1st – 2nd weeks:

- Development of the IT solution by Statistics and Bioinformatics Unit (UEB) at VHIR.
- Testing by PIs at VHIR.
- Release of the registry as a web based solution endorsed at ERN-EuroBloodNet website www.eurobloodnet.eu .
- Dissemination among ERN-EuroBloodNet members and National scientific societies for pediatric and adult hematology.

Period of COVID19 Pandemic:

- Gathering of individual data from medical centres
- Supporting medical practice
- Enabling inter-professional consultation of complex cases through ERN-EuroBloodNet channels
- Perform basic statistics

Period beyond COVID19 Pandemic:

- Observational studies on patients
- Publication of the results of observational studies
- Publication of therapeutic recommendation guidelines for the management of Covid-19 infections in hematological diseases

6. Ethics and GDPR Compliance

6.1. Background

The SARS-COV-2 pandemic disease is a public health catastrophe, due to the size of the number of infected people that pose a risk to the rest of the population. It's a health crisis with a high number of people who suffer and require health care, very often hospitable and critical. In this circumstances the general interests of the public health and, in general, of the wider public good, can face the particular interests on individual rights in favor of the public and general ones.[1]

It's very important to obtain the maximum amount of clinical information that allows a generalizable and applicable knowledge to be available, as quickly as possible, given the emergency situation (high contagiousness, disease severity, shortage of human and material resources, and lack of treatment). This fact is especially relevant in the context or rare diseases due to the lower number of patients available.

During an infectious disease outbreak there is a moral obligation to learn as much as possible as quickly as possible, in order to inform the ongoing public health response, and to allow for proper scientific evaluation of new interventions being tested. Systematic observation and data collection are essential components of emergency response measures, both to guide the management of the current outbreak and to help prevent and respond to outbreaks in the future. Even if these activities are not characterized as research for regulatory purposes, an ethical analysis should be undertaken to ensure that personal information is protected from physical, legal, psychological, and other harm. Observational studies can play a critical role in reducing morbidity and mortality and addressing the social and economic consequences caused by the outbreak.[2]

6.2. Processing of personal data

The patient's information included in the database will be pseudonymised by single codification.[3] The minimization principle of data protection is followed (i.e. only year of age is collected, no identified data is collected, and only clinical data of the health care is collected). The medical doctors at each center will have a coding table in which the code can be linked to the patient's personal information. This table will be safely guarded by the medical doctor and will never leave the center. All the information stored in the database will be pseudonymised.

6.3. Justification of the waiver of informed consent

6.3.1. Legal arguments

The primary objective of this registry is to collect data of the clinical management and outcomes of patients affected by red blood cell disorders and COVID-19 for supporting daily medical practice while enabling inter-professional consultation of complex cases. The medical doctors will share each other their personal information in order to enable this consultations without managing patient's identificative information. The processing of the patient's data is necessary for reasons of substantial public interest in the area of public health (protecting against serious cross-border threat to health and ensuring high standards of quality of health care), with general and special measures to safeguard the safety and the confidentiality of the data subject (professional secrecy and pseudonymisation).[4] Thus, obtaining informed consent for this data processing is not mandatory.

The second objective of this registry is to use the collected data in future observational studies

to describe different aspects of the illnesses and to answer scientific questions to improve the patient's health care. The processing of the patient's data is necessary for the same goals described above and for scientific and research purposes in accordance with article 89(1) of the GDPR.[5] Thus, obtaining informed consent for this data processing is also not mandatory.

6.3.2. Ethical arguments

The SARS-COV-2 pandemic disease is a clearly identified, serious and immediate threat and the requirements for consent may be waived to protect the health of the population.[6] From the guidelines CIOMS perspective there are also arguments for the waiver of the informed consent: 1- the registry has important social value, 2- the registry poses no risk to participants, 3- the objectives of the registry could not be feasible without the waiver, 4- there are an appropriate governance system for the data management.[7] It's desirable to collect all the available clinical information to guarantee the external validity of the future research. The absence of information secondary to cases in which informed consent could not be obtained could compromise the scientific value of the research and the current situation of the coronavirus pandemic is pressing.

6.4. Regulatory and Ethical Approval

6.4.1. Regulatory approval.

The Spanish Agency of Medicines and Medical Devices (AEMPS) has classified this registry as an Observational Study No Post-Authorization (No-EPA).[8] The classification will be requested from the AEMPS for future studies carried out with the registry data and all future observational research will be approved by a REC before it begins. [9]

6.4.2. Ethical approval.

The start of this registry has been approved by the VHH's REC. However, investigators should seek advice from their RECs to know if its approval is necessary before the start of the registry at their respective centers. All future observational research will be approved by a REC before it begins.

6.4.3. Governance

The registry will be localized at VHIR and the data introduced will be pseudonymised. Appropiate computing safety measures will be implemented in order to avoid the access of non-authorized people. Every medical doctor will have a password to access to the registry. The finish of the registry is open and justified while the coronavirus pandemia lasts.

There is a Scientific Committee that will evaluate the social and scientific value of future research proposals done by the medical doctors of any participating center. The protocol for every study using the registry data will be submitted to a REC. The investigational team will sign an express commitment to confidentiality. The possibility of re-identification of participants is impossible because the data used are pseudonymised. Anyway, the Scientific Committee will do a data protection impact assessment to evaluate and mitigate any possible risk of reidentification. All the information will be submitted to the REC.[7,9]

Scientific Committee functions:

- Evaluate the future research proposals with de registry data
- Evaluate future amendments of the registry

7. References

[1] Informe del Ministerio de Sanidad sobre los aspectos éticos en situaciones de pandemia: El SARS-CoV-2. Ministerio de Sanidad y Consumo de España, 2020.
[2] Guidance for Managing Ethical Issues in Infectious Disease Outbreaks. WHO, 2016.
[3] Definitions for genomic biomarkers, pharmacogenomics, pharmacogenetics, genomic data and sample coding categories. ICHTopic E15, 2007.
[4] Regulation (EU) 2016/679 of the European Parliament and of the council of 27 april 2016 on the protection of natural persons with regard to the processing of personal data and on the free movement of such data, and repealing Directive 95/46/EC (General Data Protection Regulation). Recital of consideration 54 and articles 9.2.g and 9.2.i.
[5] See also article 9.2.j of the RGPD 2016/679.
[6] Declaration of Taipei on ethical considerations regarding health databases and biobanks.WMA, 2016.
[7] International Ethical Guidelines for Health-related Research Involving Humans, Fourth Edition. Geneva. Council for International Organizations of Medical Sciences (CIOMS); 2016.
[8] Orden SAS/3470/2009, de 16 de diciembre, por la que se publican las directrices sobre estudios posautorización de tipo observacional para medicamentos de uso humano.
[9] Ley Orgánica 3/2018, *de 5 de diciembre, de Protección de Datos Personales y garantía de los derechos digitales*. Disposición adicional 17.
